# Retrospective Analysis of Efficacy and Toxicity of Stereotactic Body Radiotherapy and Surgical Resection of Adrenal Metastases from Solid Tumors

**DOI:** 10.3390/cancers16152655

**Published:** 2024-07-26

**Authors:** Jamie Lütscher, Hans Gelpke, Adrian Zehnder, Laetitia Mauti, Christian Padevit, Hubert John, Nidar Batifi, Daniel Rudolf Zwahlen, Robert Förster, Christina Schröder

**Affiliations:** 1 Department of Radiation Oncology, Cantonal Hospital Winterthur, 8401 Winterthur, Switzerland; 2 Department of Visceral and Thoracic Surgery, Cantonal Hospital Winterthur, 8401 Winterthur, Switzerland; 3 Department of Medical Oncology, Cantonal Hospital Winterthur, 8401 Winterthur, Switzerland; 4 Department of Urology, Cantonal Hospital Winterthur, 8401 Winterthur, Switzerland; 5 Department of Radiation Oncology, Inselspital, Bern University Hospital, University of Bern, 3010 Bern, Switzerland

**Keywords:** adrenalectomy, stereotactic body radiation therapy, adrenal metastases, local control, progression free survival, overall survival, complication rate

## Abstract

**Simple Summary:**

The adrenal glands represent a frequent localization area for metastases of various primary tumors. The standard of care for treating adrenal metastases is currently surgical resection, but radiotherapy is becoming a feasible and well-tolerated treatment alternative. However, the literature shows contradictory results regarding the optimal local treatment for adrenal metastases. This study analyzed oncological outcomes (local control, progression free survival, and overall survival) and complication rates after stereotactic body radiotherapy or surgical resection of adrenal metastases to further asses the benefits of stereotactic body radiotherapy. To our knowledge, this analysis is the first direct comparison of stereotactic body radiotherapy and adrenalectomy for adrenal metastases.

**Abstract:**

Background: This single-center retrospective study aimed to evaluate the efficacy and toxicity profiles of stereotactic body radiotherapy (SBRT) and surgical resection in patients with adrenal metastases originating from solid tumors. Methods/Materials: Patients with advanced tumor conditions or comorbidities typically received SBRT, whereas those considered physically fit underwent standard surgical treatment. Endpoints included local control (LC), progression free survival (PFS), overall survival (OS), and complication rates (CR). Results: 41 patients with 48 adrenal metastases were included, with 27 (65.9%) patients receiving SBRT and 14 (34.1%) patients undergoing adrenalectomy. One- and two-year LC values were 100% for both periods after adrenalectomy, and 70.0% and 52.5% after SBRT (*p* = 0.001). PFS showed values of 40.2% and 32.1% at one and two years after adrenalectomy and of 10.6% for both periods after SBRT (*p* = 0.223). OS was 83.3% both one and two years after surgery and 67.0% and 40.2% after SBRT (*p* = 0.031). There was no statistically significant difference between the two groups regarding acute complications (*p* = 0.123). Conclusion: Despite potential confounders, adrenalectomy exhibited statistically significant superior LC and OS compared to SBRT in managing adrenal metastases, while both treatment methods displayed acceptable toxicity profiles. However, patient selection bias must be taken into account when directly comparing the two therapy modalities. Nevertheless, the study provides new and important results for the scientific and medical communities regarding oncological outcomes after SBRT or surgical resection of adrenal metastases.

## 1. Introduction

The adrenal glands are common sites for metastases originating from various primary tumors [1], which can be observed in 15–35% of oncological cases [2], primarily due to their abundant blood supply [3]. Lung, breast, colorectal cancers, melanoma, and renal cell carcinoma are frequent primaries [4]. Patients with adrenal metastases may present as asymptomatic or experience symptoms like back pain or adrenal insufficiency [2,5].

In oligometastatic disease (OMD), partially defined by a limit of five metastases to a maximum of three organs [6], local treatment of adrenal metastases through interventions such as surgery, radiotherapy, or interventional radiology techniques is effective and safe and can be associated with a prolonged overall survival (OS) compared to the natural course of the disease [1,2,5,7,8,9,10]. Surgical resection remains the standard, although other local therapies as well as immunotherapy and targeted therapy have shown promising results [2,11,12]. Stereotactic body radiotherapy (SBRT) is a feasible and well-tolerated treatment alternative, particularly in comorbid patients [2,5,13,14]. In our cancer center, patients with advanced tumor conditions or comorbidities were typically administered SBRT, whereas those deemed physically fit underwent standard surgical treatment. 

However, data regarding the optimal treatment method for adrenal metastases with respect to oncological outcomes vary. Hatano et al. [1] reported one- and two-year OS of 92% and 83% following adrenalectomy, while Yuste et al. [15] showed OS rates of 76% and 55.8%, respectively, after SBRT. Van Vliet et al. [16] found no significant difference in OS or complication rates between surgical resection and SBRT, but noted higher rates of local control (LC) and progression free survival (PFS) with SBRT in a study of 97 patients. Franzese et al. [2] suggested that variations in oncological outcomes may be influenced by factors such as the number and sites of metastases [11,12]. 

Due to these contradictory results addressing optimal local treatment for adrenal metastases, we conducted a retrospective analysis comparing the effectiveness of SBRT versus surgical resection in terms of local control, progression free survival, overall survival, and complication rates at our cancer center.

## 2. Materials and Methods

### 2.1. Study Design and Endpoints

We conducted a retrospective study based on a database analysis focusing on patients with solid tumors metastasized to the adrenal glands at the Cantonal Hospital Winterthur, Winterthur, Switzerland, between 2013 and 2023. Adrenal metastases were either treated by SBRT (group A) or surgery (group B). In our hospital, fitter patients were typically treated with standard surgical procedures, while unfit patients with multiple comorbidities or advanced tumor diseases were more likely to undergo SBRT. The primary endpoints included local control (LC), progression free survival (PFS), overall survival (OS), and complication rates (CR), which encompassed hospitalization time, analyzed from the initiation of local treatment to event. LC was defined as time to recurrence or progression of the treated adrenal metastasis. PFS and OS were calculated from start of local therapy for an adrenal metastasis until either local or systemic progression or death (PFS), or until death of any cause (OS). Severe complications were defined by toxicity grade ≥2 according to the Common Terminology Criteria for Adverse Events (CTCAE), version 5.0. 

### 2.2. Radiotherapy Planning

Patients received LINAC-based SBRT either by 4D-CT or with the breath-hold technique. For 4D-CT patients, a gross tumor volume (GTV) was contoured in all breathing phases using all imaging data available (PET-CT, MRI) creating an internal target volume (ITV). For breath-hold patients, GTV was contoured without ITV creation. No clinical target volume (CTV) margin was applied, but a margin of 5–10 mm was added to form the planning target volume (PTV). The prescription dose was normalized to the 80% isodose line (IDL), except for a few cases with homogeneous prescription (100% IDL). SBRT was delivered with image-guided intensity modulated radiotherapy (IMRT) or volumetric modulated arc therapy (VMAT) using daily cone beam CT (CBCT). No fiducials were implanted. Additional treatment related characteristics are shown in Section 3.1.

### 2.3. Surgery Technique

Adrenalectomy was either performed laparoscopically, with robot-assisted surgery (da Vinci) or with an open technique (laparotomy). Both patients who underwent single and extended adrenalectomies were included in this study. Additional treatment related characteristics are shown in Section 3.1. 

### 2.4. Statistical Analysis and Ethics

The statistical analysis was performed using the statistical software “Statistical Package for Social Science” (SPSS) by IBM (version 29.0.0.0 (241); International Business Machines Corporation IBM, Armonk, NY, USA). The biologically equivalent dose (BED), which embodies an established measure to compare the biological effects of the different radiation dose fractionation schedules, was calculated with an alpha/beta value of 10 Gy. Descriptive statistics were obtained using the Kaplan–Meier method as well as a log-rank test and Pearson’s chi-square test for group comparison. The level of significance was defined as α = 0.05. The collection of data was approved by the local Ethics Committee (reference number BASEC 2020-02112), and anonymity was guaranteed by encoding the data.

## 3. Results

### 3.1. Patient and Treatment Characteristics

We identified 41 patients with metastasized solid tumors and 48 adrenal metastases, of which 27 patients (65.9%) were treated by SBRT (group A) and 14 patients (34.1%) underwent surgery (group B). A total of seven patients (17.1%) were treated for bilateral adrenal metastases, resulting in 31 courses of adrenal SBRT within twenty-seven patients, and 17 surgical resections within fourteen patients. Of the seven patients treated bilaterally, three received bilateral SBRT, three bilateral resection, and one patient received surgery to one side and SBRT to the other. 

The median age of the patients was 62 years (range 30–78 years) at first diagnosis and 64 years (range 32–81 years) at the time of first local treatment for adrenal metastases. The majority of the patients suffered from lung cancer (*n* = 28, 68.3%) as their primary diagnosis, along with several other cancer entities (*n* = 13, 31.7%). The median follow-up was 11.3 months for the radiotherapy cohort and 36.9 months for the surgical cohort.

SBRT was predominantly performed over five fractions to a dose of 30–40 Gy at 80% isodose line (*n* = 24, 77.4%) using LINAC-based motion management (4D-CT, deep inspiration breath hold (DIBH), expiration breath hold (EBH)). The median diameter of the adrenal metastases was measured at 40 mm (range 18.0–172.0) in the radiotherapy group and 19 mm (range 1.0–77.0) in the surgically treated group. The median value of the gross tumor volume (GTV) was 34.0 cc (range 4.0–949.2 cc). Adrenalectomy was performed either laparoscopically (*n* = 8, 47.1%), openly (*n* = 6, 35.3%), or robot-assisted using the da Vinci method (*n* = 3, 17.6%). In most cases, a single adrenalectomy was performed (*n* = 13, 76.5%) as compared to an extended adrenalectomy (*n* = 5, 29.4%). Additional patient and treatment related characteristics are shown in Table 1 and Table 2.

### 3.2. Local Control

**Figure 1:**
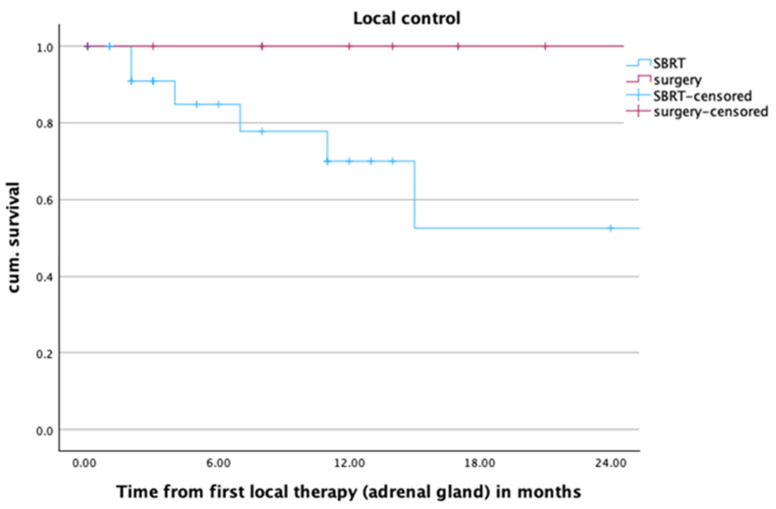
Local control of adrenal metastases (*n* = 48) after radiotherapy or surgery within the first two years after local treatment of adrenal metastases, *p* = 0.001.

One- and two-year local control rates in the overall cohort were 84.4% and 78.7%. For lesions treated with SBRT, those values were 70.0% and 52.5%, as opposed to 100% and 100% for lesions treated with adrenalectomy. There were overall eight local treatment failures in the SBRT group. Six of them developed infield, one presented as a marginal treatment failure, and one is unknown. Two of them were treated with salvage adrenalectomy. Salvage SBRT was only used in one patient after R1 resection. All patients who showed a local failure also suffered from systemic tumor progress. Figure 1 shows the Kaplan–Meier curve for local control after SBRT and adrenalectomy. The difference between the treatment groups was statistically significant (*p* = 0.001).

Upon closer examination of the sizes of adrenal metastases, the study revealed that local control was particularly worse for large metastases (>2 cm) treated by SBRT. Smaller metastases (<2 cm) or surgically treated metastases exhibited excellent local control within the first two years after local treatment (LC in patients with metastases size < 2 cm: SBRT 100%, surgery 100%; LC in patients with metastases size > 2 cm: SBRT 47.8%, surgery 100%; *p* = 0.004).

### 3.3. Distant Progression

**Figure 2:**
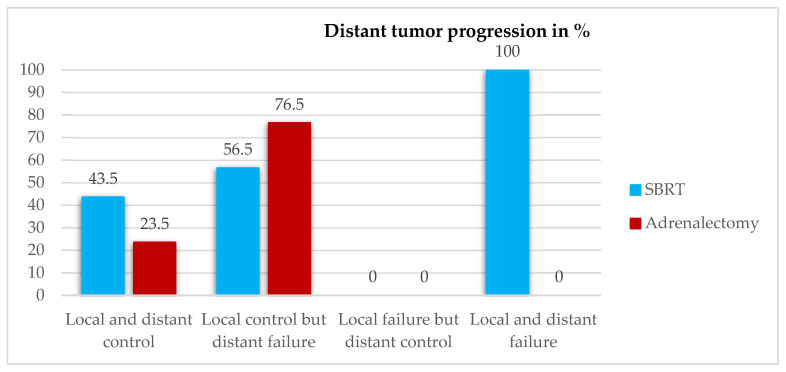
Distant tumor progression in relation to local control two years after local treatment.

Two years after SBRT, distant systemic tumor progression could be observed in all patients with local failure and in 56.5% (*n* = 13) of the patients with local control. When it comes to adrenalectomy, distant failure (*n* = 13, 76.5%) could only be observed in patients with complete LC, as no local failure was seen. More information regarding the relation between local control and distant tumor progression can be found in Figure 2.

### 3.4. Progression Free Survival

**Figure 3:**
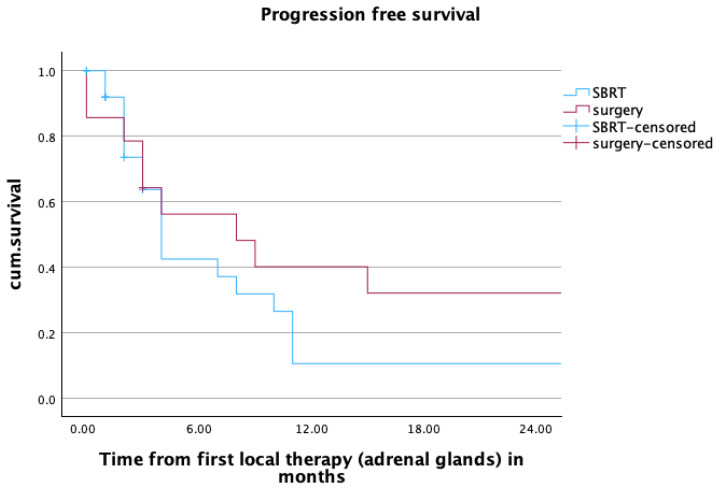
Progression free survival of patients (*n* = 41) after radiotherapy or surgery within the first two years after local treatment of adrenal metastases. mPFS SBRT vs. surgery: 4 vs. 8 months, *p* = 0.223.

One- and two-year PFS for patients (*n* = 41) in the overall cohort was 22.5% and 19.3%, respectively. In patients receiving SBRT, those values were 10.6% for both one- and two-year PFS, and 40.2% and 32.1% for patients undergoing adrenalectomy. The estimated median PFS was 4 months for patients in the SBRT group, and 8 months for patients treated with surgery. The difference between the treatment groups was not statistically significant (*p* = 0.223). More information about the PFS can be found in Figure 3.

### 3.5. Overall Survival

**Figure 4:**
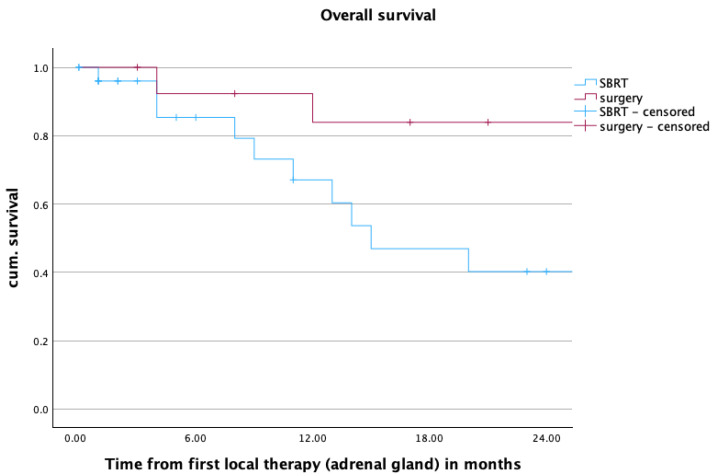
Overall survival over the first two years after radiotherapy or surgery, mOS SBRT vs. surgery: 15 vs. 97 months, *p* = 0.031.

In the overall cohort, one- and two-year OS was 73.9% and 58.9%. For patients treated with SBRT, those values were 67.0% and 40.2%. For patients treated with surgery, those values were 83.9% for both one- and two-year OS. The estimated median OS was 15 months in the SBRT group and 97 months in the surgery group. The difference between the treatment groups was statistically significant (*p* = 0.031). Upon closer examination of age dependency, deceased patients treated with SBRT exhibited a comparable age profile to those who underwent surgery, but demonstrated a shorter overall survival. While the median age at local treatment time in the SBRT group was 64.0 years (range: 54–70) with a median OS of 8 months (range: 1–14) after local treatment, the median age at local treatment time in the adrenalectomy group was 66 years (range 63–69) with a median OS of 20.5 months (range 12–29) after local treatment. Figure 4 shows the Kaplan–Meier curve for OS after SBRT and adrenalectomy.

### 3.6. Hospitalization Time and Toxicity

The median time of hospitalization was 4 days (range 2–22 days) in the surgery group, while none of the patients with SBRT were hospitalized for treatment.

There was no statistically significant difference between the two groups regarding acute complications (*p* = 0.123). In the surgery group, there were four ≥G2 acute complications (23.5%). One patient suffered from a G2 wound infection, and another had an accidental opening of the bowel with fistula and intra-abdominal abscess (G3). Two further patients suffered from G4 complications with circulatory instability due to a central pulmonary embolism and high intraoperative blood loss with SIRS. Both patients with G4 complications had a more extended surgery. There were two ≥G2 acute toxicities in the radiotherapy group (6.5%). One patient reported G2 abdominal pain. The other patient suffered from G5 acute toxicity. This patient had a large (950 cc) adrenal metastasis that infiltrated multiple structures including the bowel. It was deemed unsuitable for resection and the patient received radiotherapy with 5 × 5 Gy homogenous. A week after the end of radiotherapy, the patient presented with a superinfection of the necrotic adrenal metastasis, resulting in a fistula between the remains of the irradiated adrenal metastasis and the left colon flexure two months later. The patient underwent extensive resection, including adrenalectomy and hemicolectomy, but died of sepsis related to colon anastomosis insufficiency four months after radiotherapy. 

There were no ≥G2 late complications in either group, except for 4 patients with adrenal insufficiency after bilateral adrenalectomy (three after initial surgery, and one patient after bilateral salvage adrenalectomy).

## 4. Discussion

This study analyzed oncological outcomes (LC, PFS, OS) and complication rates after SBRT or surgical resection of adrenal metastases. SBRT showed statistically worse LC and OS compared to adrenalectomy, along with worse PFS, though not statistically significant. To our knowledge, this analysis is the first direct comparison of SBRT and adrenalectomy for adrenal metastases. 

To better understand the worse oncological outcomes in SBRT patients compared to surgery recipients, we initially compared treatment accuracy with the existing literature data. 

Most studies on SBRT generally report one- to two-year LC at 76–85.9% and 72.5–74% [2,15,17], one- to two-year PFS at 35.4–45% and 22.3–30% [15,18], and one- to two-year OS at 76% and 55.8%, respectively [15]. Our study yielded slightly inferior results, potentially influenced by the lower BED values chosen (BED_10_ of 28–65.63 Gy) than generally recommended in the literature (BED_10_ of 72–116 Gy) [19,20,21,22]. Furthermore, the relative effectiveness of SBRT was assessed in a patient population not suitable for surgery due to comorbidities or advanced tumor disease, which might also explain the slightly poorer outcomes compared to the literature. Nevertheless, in most studies known to date, no distinction was made between SBRT as a primary therapy option and SBRT as an alternative therapy for non-operable patients.

Regarding adrenalectomy, little data is available concerning LC and PFS, making comparison of data challenging. However, comparable OS values to the existing literature one to two years after adrenalectomy were found (93.3–86.2% vs. 92–83%) [1,23,24]. Laparoscopic surgery demonstrated similar oncological outcomes to open surgery, as previous studies have highlighted [1,25,26]. Therefore, both treatment methods align reasonably with the findings in the literature.

When comparing the SBRT and adrenalectomy patient groups, differences in oncological outcomes arose. SBRT patients exhibited poorer outcomes, possibly due to specific patient selection regarding the patient’s underlying disease state and performance status: more SBRT patients had initial metastasis (group A: 43.9%; group B: 22.0%) and active tumors during treatment (group A: 12.2%; group B: 0.0%) compared to surgery patients. Previous studies have identified this metastatic presentation as a prognostic factor for better OS and PFS [1,2,15,23,27]. Additionally, SBRT patients received more prior systemic therapies (group A: 36.6% immunotherapy, 19.5% chemotherapy; group B: 0.0% immunotherapy, 22.0% chemotherapy) and showed a larger median size of the adrenal metastases (group A: 4 cm; group B: 1.9 cm) than surgery recipients, again referring to a poorer prognostic index in the SBRT group. Hatano et al. [1] considered a tumor size ≥ 4 cm as a significant risk factor for progression. Our study revealed a distinct decline in local control following SBRT for metastases ≥ 2 cm, whereas adrenalectomy did not show such an effect. Therefore, unfit patients with multiple metastases and limited systemic options were more likely to receive SBRT, while fitter patients often underwent surgery, indicating significant patient selection bias. This specific patient selection bias likely influenced the study and must therefore be considered when directly comparing the two therapy modalities. However, apart from the direct comparison of these two treatment options, the study still provides new and important results for the scientific and medical communities regarding oncological outcomes after SBRT or adrenalectomy. Therefore, the data should be interpreted as reference values after SBRT or surgery without directly comparing them. Additionally, previous studies have suggested that prior treatments correlate with enhanced local tumor control, leading to improved systemic disease control (OMD concept) [28], which was affirmed in our study: patients with local failure experienced systemic tumor progression, contrasting those with complete local control. 

In our analysis, overall toxicity rates were acceptable in both treatment groups, despite longer hospitalization stays for surgically treated patients. However, SBRT showed less ≥G2 toxicity incidence (6.5%) compared to surgery (23.5%). The literature supports SBRT’s efficacy, with mostly grade 1 toxicities [15,22,29]. However, one patient in the SBRT group died due to abscessing necrosis of the treated adrenal gland, leading to surgery and subsequent complications. The treatment dose for this patient was conservative (5 × 5 Gy homogeneous), but the large size of the treated adrenal metastasis (950 cc) limited surgical resection. Debate exists on whether the metastasis size influenced toxicity rates due to organ exposure [1,30]. The literature also debates surgical resection safety for large adrenal metastases [31,32], with some studies reporting no major complications and others noting common postoperative issues such as ileus, gastroparesis, wound infections, pneumonia and arrhythmias [33]. This increased complication rate after surgery must be balanced against its high oncological control compared to SBRT, which may have fewer toxicities, but also lower tumor control rates.

This study has limitations impacting results, which include its retrospective design, the small patient population, and the heterogenic cohort. Variants in histology, systemic therapy types, radiation treatment, and surgery could also influence outcomes. Patients receiving SBRT had poorer predictive values and performance status compared to surgery patients, leading to selection bias, which must be considered. Further prospective studies are needed to mitigate these limitations.

## 5. Conclusions

Although adrenalectomy exhibited statistically significant superior LC and OS compared to SBRT in this cohort, longer hospitalization stays were necessary in the surgical patient group. Both treatment methods displayed favorable results regarding oncological outcomes with acceptable toxicity profiles compared to the literature. Nevertheless, specific patient selection influenced the outcome: unfit patients with multiple metastases and less viable systemic options were more likely to be treated with radiotherapy, whereas fitter and less tumor-burdened patients more often underwent surgery. This patient selection must be considered when directly comparing the two treatment modalities. Nevertheless, this study contains new and important results for the scientific and medical communities and underscores the importance of performing randomized trials to eliminate bias and obtain tailored treatment decisions based on efficacy, toxicity, and individual patient considerations. Additional prospective studies are necessary to confirm this analysis.

## Figures and Tables

**Table 1:** Patient characteristics per patient (*n* = 41, 100%).

	SBRT		Adrenalectomy		All	
	*n*	%	*n*	%	*n*	%
*Sex*						
Female	8	19.5	4	9.8	12	29.3
Male	19	46.3	10	24.4	29	70.7
*Primary Entity*						
NSCLC Adeno	10	24.4	9	22.0	19	46.3
NSCLC other ^1^	6	14.6	1	2.4	7	17.1
SCLC	2	4.9	0	0.0	2	4.9
Adenocarcinoma of the GI tract	1	2.4	2	4.9	3	7.3
Renal cell carcinoma	1	2.4	2	4.9	3	7.3
Other ^2^	7	17.1	0	0.0	7	17.1
*M1 at initial diagnosis*						
No	8	19.5	5	12.2	13	31.7
Yes	18	43.9	9	22.0	27	65.9
Unknown	1	2.4	0	0.0	1	2.4
*Solitary adrenal metastasis*						
Yes	7	17.1	6	14.6	13	31.7
No	20	48.8	8	19.5	28	68.3
*Active tumor disease at local treatment time*						
Yes	5	12.2	0	0.0	5	12.2
No	22	53.7	14	34.1	36	87.8
*Line of systemic therapy at local adrenal treatment*						
None	2	4.9	2	4.9	4	9.8
1	16	39.0	10	24.4	26	63.4
2	8	19.5	1	2.4	9	22.0
3	1	2.4	1	2.4	2	4.9
*Type of systemic therapy up to local adrenal treatment*						
None	2	4.9	2	4.9	4	9.8
Chemotherapy	8	19.5	9	22.0	17	41.5
Regimen including Immunotherapy	15	36.6	0	0.0	15	36.6
Regimen including Targeted Therapy	1	2.4	3	7.3	4	9.8
Endocrine therapy	1	2.4	0	0.0	1	2.4
*Systemic treatment beyond progression*						
Yes	5	12.2	0	0.0	5	12.2
No	22	53.7	14	34.1	36	87.9
	**All**					
	**Median**			**Range**		
Age at first diagnosis (years)	62.0			30–78		
Age at first adrenal treatment (years)	64.0			32–81		
Duration of systemic treatment beyond progression after local treatment (months)	7.0			2–36		
Time to next systemic treatment after local treatment (months)	10.0			5–33		

^1^ NSCLC other histology: squamous cell carcinoma (2), neuroendocrine tumor (1), giant cell carcinoma (1), pleomorphic malignant neoplasm (1), adenosquamous carcinoma (1). ^2^ Other primary entities: pancreatic adenocarcinoma (2), urothelial carcinoma (1), mesothelioma (1), prostate cancer (1), CUP (1), esophageal adenocarcinoma (1), lingual carcinoma (1).

**Table 2:** Treatment characteristics per adrenal metastasis (*n* = 48, 100%).

Treatment Characteristics	Amount	
	*n*	%
*Surgery Technique*		
Open	6	35.3
Laparoscopically	8	47.1
da Vinci	3	17.6
*Extent of surgery*		
Adrenalectomy only	13	76.5
Extended adrenalectomy	5	29.4
R0	14	82.4
R1	3	17.6
*Bilateral treatment (per patient)*		
Bilateral adrenalectomy	3	7.3
Bilateral SBRT	3	7.3
Both adrenalectomy and SBRT	1	2.4
*Salvage therapy*		
Salvage adrenalectomy	2	4.2
Salvage SBRT	1	2.1
*Fractionation*		
5 × 5 Gy @ 100%	4	12.9
5 × 6 Gy @ 80%	13	41.9
5 × 7 Gy @ 80%	6	19.4
5 × 8 Gy @ 80%	3	9.7
Other ^1^	5	16.1
*Motion Management*		
4D	20	64.5
EBH	6	19.4
IBH	3	9.7
none	2	6.5
	**Median**	**Range**
*Size of metastases (diameter in mm)*		
Group A (SBRT)	40	18.0–172.0
Group B (adrenalectomy)	19	1.0–77.0
*SBRT-target volume (cc)*		
GTV	34	4.0–949.2
PTV	79.9	23.0–1328.0
*Biological Equivalent Dose (BED Gy_10_)*	47.7	28.0–65.63

^1^ Other fractionations: 5 × 4 Gy homogeneous, (2), 5 × 7.5 Gy @ 80% (2), 13 × 3 Gy homogeneous (2).

## Data Availability

Data available on request due to restrictions (ethical reasons).
